# Prevalence of feline haemoplasma in cats in Denmark

**DOI:** 10.1186/s13028-016-0260-1

**Published:** 2016-11-10

**Authors:** Maja Benedicte Rosenqvist, Ann-Katrine Helene Meilstrup, Jesper Larsen, John Elmerdahl Olsen, Asger Lundorff Jensen, Line Elnif Thomsen

**Affiliations:** 1 Department of Veterinary Disease Biology, Faculty of Health and Medical Sciences, University of Copenhagen, Stigboejlen 4, 1870 Frederiksberg C, Denmark; 2 Department of Veterinary Clinical and Animal Sciences, Faculty of Health and Medical Sciences, University of Copenhagen, Dyrlaegevej 16, 1870 Frederiksberg C, Denmark; 3 Statens Serum Institut, Artillerivej 5, 2300 Copenhagen S, Denmark

**Keywords:** Feline haemoplasma, Prevalence, Real-time PCR, Conventional PCR, Risk factors

## Abstract

**Background:**

Infections with the three feline haemotropic mycoplasmas *Mycoplasma haemofelis*, *Candidatus* Mycoplasma haemominutum and *Candidatus* Mycoplasma turicensis cause feline infectious anemia. The purpose of this study was to investigate the prevalence of carriage of feline haemoplasma in Danish cats in different age groups. The presence was detected by a conventional polymerase chain reaction (PCR) assay on blood samples as well as by real-time PCR (RT-PCR).

**Results:**

The study revealed a prevalence of 14.9% *Candidatus* Mycoplasma haemominutum positive cats and 1.5% *Mycoplasma haemofelis* positive cats. No cats were found positive for *Candidatus* Mycoplasma turicensis. The results showed a statistically significant higher prevalence in older (>8 years) cats compared to younger cats and a higher prevalence among domestic cats compared to purebred cats. As part of this study, we developed a cloning strategy to obtain Danish positive controls of haemoplasma 16S rRNA.

**Conclusion:**

From convenience-sampled cats in Denmark, we found that 16.4% were carriers of feline haemotropic mycoplasmas. Haemoplasma was mostly found in older and domestic cats. The prevalence found in Denmark is similar to that found in several other European countries.

**Electronic supplementary material:**

The online version of this article (doi:10.1186/s13028-016-0260-1) contains supplementary material, which is available to authorized users.

## Background

Feline haemotropic mycoplasmas (haemoplasmas) are small epierythrocytic Gram-negative bacteria, which can only survive by parasitism of erythrocytes [1] and which cause feline infectious anemia. The feline haemoplasma group consists of at least three species, *Mycoplasma haemofelis* (Mhf), *Candidatus* Mycoplasma haemominutum (CMhm) and *Candidatus* Mycoplasma turicensis (CMt) [1–3]. Infections with these three species differ in severity with Mhf inducing the most severe symptoms such as anorexia, depression, fever and anemia; whereas the CMhm and CMt infected cats rarely show any clinical signs [4–6]. CMhm usually require co-infection with another haemoplasma or immune-compromised conditions to cause disease and the pathogenic potential of CMt most likely also depends on cofactors [7–9].

Feline haemoplasmas are widely distributed in pet cats throughout the world, although the prevalence varies geographically [9–13]. These variations may be due to differences in climate as studies have shown a correlation between haemoplasma prevalence and warmer climate [9–11, 14]. The higher prevalence is suggested to be due to presence of a higher number of blood-sucking arthropods, which are suspected to be vectors for haemoplasmas [12, 15]. The risk of infection has also been associated with several other factors, such as age, gender and breed [10–12].

Haemoplasmas have never been cultured in vitro and identification is usually based on cytological examination of blood smears [6]. However, this method has proven inefficient due to low sensitivity and specificity resulting in false negative results. Development of PCR based methods has increased the sensitivity of identification of the organisms in blood samples [9, 12, 16–18]. Feline infectious anemia was initially reported in Denmark in 1974, but the prevalence is unknown [18]. In this study, we used PCR methods to determine the prevalence of feline haemoplasmas in the blood of convenience samples of cats from Denmark and evaluated possible associations to breed, age, gender and symptoms.

## Methods

### Collection of blood samples and clinical information

Seventy-three blood samples, representing blood from 67 cats sampled during 2007–2008 were used. Forty-six of the samples had been collected from patients at the University Hospital for Companion Animals, Faculty of Health and Medical Sciences, University of Copenhagen. The remaining 27 blood samples were collected at veterinary clinics throughout Denmark. The blood samples were collected from both diseased cats and cats attending the clinics for routine health checks. From four of the cats from the University Hospital we had samples in duplicate or triplicate. These samples were used as controls for the PCR reaction to confirm that we obtained identical results on repeated testing. For 62 cats, information on age, breed and gender was available. Anamnesis and clinical findings were available for all cats admitted to the University Hospital and from ten samples from the other clinics. The blood samples were obtained by venal puncture and stored in 2.5 ml EDTA-vials at −80 °C. Blood samples obtained from the University Hospital were all subjected to haematological examination using the ADVIA 120 haematology system including erythrocyte count, haemoglobin-, haematocrit- and mean cell volume (MCV) values.

### DNA extraction

Extraction of total DNA from the blood samples was done using the DNeasy® Blood and Tissue kit (Qiagen, Denmark). 100 µl EDTA blood and 100 µl PBS was used for each DNA purification.

### PCR amplification and cloning of 16S rRNA from haemoplasma

Conventional PCR: 0.8 µM of each primer M-forward: 5′ ACGAAAGTCTGATGGAGCAATA 3′ and M-reverse: 5′ ACGCCCAATAAATCCGRATAAT 3′ [5]. PCR was performed using the Thermo Hybaid PCR express machine using the following conditions, Segment 1: 94 °C 1 min, Segment 2: 94 °C 1 min, 60 °C 1 min, 72 °C 30 min at 45 cycles and Segment 3: 72 °C for 10 min.

RT-PCR: 0.3 µM of the HPLC purified primers, M-forward: 5′ ACGAAAGTCTGATGGAGCAATA 3′ and M-reverse: 5′ ACGCCCAATAAATCCGRATAAT 3′ [5]. RT-PCR was performed using the Mx30000P® and Brilliant® SYBR Green QPCR master mix (Stratagene, California, USA) essentially as described by the manufacturer. The RT-PCR reaction was run under the following conditions: Segment 1: Initial denaturation 95 °C 10 min, Segment 2: 95 °C 30 s, 60 °C 1 min, 72 °C 30 s, for 40 cycles, Segment 3: 95 °C 1 min, ramp down to 55 °C and ramp up from 55 to 95 °C. Dissociation temperatures were 77.4 °C for CMhm, 78.0 °C for Mhf and 79.5 °C for CMt. Primers for both conventional and RT-PCR amplify products of 193 bp for CMhm and 170 bp for both Mhf and CMt.

DNA from Mhf, CMhm and CMt was used as positive controls for the PCR methods [19]. The samples were used as positive controls in the initial analysis to confirm that the used PCR and RT-PCR conditions could produce the expected PCR products. After confirmation, the 16S rRNA PCR fragments from the first haemoplasma-positive blood samples (Mhf and CMhm) were used and cloned using the TOPO TA Cloning® Kit as described by the manufacturer (Invitrogen, Denmark). Since we found no CMt positive cats, we also cloned the CMt 16S rRNA. All cloned fragments were sequenced, confirming that the DNA was of the expected sequence. Plasmid purification was done using the QIAprep® spin miniprep kit from Qiagen. Sequencing of the cloned samples was done using 0.8 µM of each of the primers M13-forward: 5′ GTAAAACGACGGCCAGT 3′ and M13-reverse: 5′ AACAGCTATGACCATG 3′. These plasmids were used as positive controls in the PCR and RT-PCR analysis of the blood samples using the M-forward and M-reverse primers as described under the two PCR methods.

### DNA sequencing

DNA sequencing of PCR products was performed on all positive samples (DNA Technology A/S). The sequence identity was confirmed by alignment to identity to GenBank accession number EU170604 and EU839985 (Mhf), EU839977 (CMt) and to EU839978 (CMhm).

### Statistical analysis

Statistical analysis on the available information on gender, age and breed was done using GraphPad Prism 5 (https://www.graphpad.com). Fischer´s exact test was used to test for association between presence of Mhf/CMhm and age, gender and breed. Mann–Whitney U-test was used to test for significant differences in the haematological variables.

## Results

### Prevalence of carriage of feline haemoplasma species

Blood samples collected from 67 Danish cats were investigated to estimate the prevalence of carriage of the three haemoplasmas, CMhm, Mhf and CMt. For each cat, information about breed, gender, age and symptoms/diagnosis were obtained when possible (Additional file 1: Table S1). The haemoplasmas are non-culturable in vitro and accurate diagnosis is currently reliant on detection of bacterial DNA using PCR assays [20]. Eleven animals were found to be positive among the 67 cats tested (Table 1). Ten cats were positive using both conventional and RT-PCR while one additional cat was positive by RT-PCR only. Carriage of multiple haemoplasma species was not detected. From four PCR negative cats [cats 4, 5, 10, 15 (Additional file 1: Table S1)], more than one sample was analyzed [samples 51, 11, 38, 29, 41 and 55 (Additional file 1: Table S1)], and all samples yielded the same negative result upon repeated testing.

**Table 1:** Haemoplasma prevalence in blood from 67 cats in Denmark determined by PCR and RT-PCR

Species	Positive by PCR	Prevalence PCR (%)	95% CI	Positive by RT-PCR	Prevalence RT-PCR (%)	95% CI
Mhf	1	1.5	0–4.4	1	1.5	0–4.4
CMhm	9	13.4	5.2–21.6	10	14.9	6.4–23.4
CMt	0			0		
Total	10	14.9	6.4–23.4	11	16.4	7.5–25.3

*Mhf Mycoplasma haemofelis*, *CMhm Candidatus* Mycoplasma haemominutum, *CMt Candidatus* Mycoplasma turicensis, *CI* confidence interval

### Confirmation of PCR positive samples

Based on fragment sizes, 10 positive samples were judged as harboring CMhm, while one fragment corresponded to Mhf/CMt. Since the PCR products of Mhf and CMt positive cats are of equal size, all PCR products were sequenced to confirm the results and discriminate between these two species. The sequencing revealed that the 10 CMhm positive samples indeed contained DNA from CMhm. Seven had 100% identity (E value: 3e^−74^) to GenBank accession number EU170604 and three had 97–100% identity (E value: 3e^−69^) to EU839985. The one sample identified as Mhf/CMt showed 98% identity (E value: 3e^−58^) to Mhf EU839978. We found no samples with homology to CMt among the Mhf/CMt positive samples, corresponding to 0% prevalence with a confidence interval between 0 and 4.7%. Control DNA from CMt did result in the expected sequence of the PCR fragment, showing that the primers did amplify the CMt DNA.

### Association between presence of haemoplasma and clinical signs

The statistical tests for significant differences in haematological values in cats being CMhm positive/negative showed no significant differences (P > 0.5). One cat (Additional file 1: Table S1, no. 73) which was positive for Mhf, suffered from dehydration, weight loss and anorexia and the haematology of this cat showed abnormal values. Haemoglobin, haematocrit and total erythrocyte counts were decreased, whereas MCV was increased compared to normal reference interval (Table 2). The blood smear showed several epierythrocytic organisms on every erythrocyte, anisocytosis, polychromasia and regeneration with reticulocytes and metarubricytes. The smear also contained lymphoblasts.

**Table 2:** Haematology results for Mhf positive cat no. 73

Parameter	Value	Reference interval
Total erythrocyte count (billion/L)	1.49	5–10
Haemoglobin (mmol/L)	2.00	5–9.3
Haematocrit (L/L)	0.09	0.24–0.45
Mean cell volume (MCV) (fL)	60.4	40–57

### Association between carriage of haemoplasma and age, breed and sex

The univariable analyses for risk factors showed significantly higher carriage frequency in cats ≥ 8 years old compared to younger cats (P < 0.05), and a higher prevalence among domestic cats compared to purebred cats (P < 0.05). One of the cats (Additional file 1: Table S1, no. 42) was diagnosed as feline immunodeficiency virus (FIV) positive and was also infected with CMhm. Among males, 23.5% (8/34) carried haemoplasma species, whereas only 9.4% (3/32) of the females were positive. Although, prevalence in males seemed higher than in females, the difference was not statistically significant (P > 0.05).

## Discussion

The current study provides the first prevalence estimate of carriage of feline haemoplasma species in Denmark. The prevalence of CMhm and Mhf infections was 14.9 and 1.5% respectively when using RT-PCR. One sample, being positive by RT-PCR, was not identified as CMhm when using conventional PCR. Previous studies have shown a similar prevalence in other European countries [9, 19, 20]. The study was performed by the use of a convenience-sampled cat population. The cats included were examined by a veterinarian and represented both diseased cats and cats attending the clinic for routine health checks. This corresponds to the approach used in other studies [11, 13].

The most frequent haemoplasma found in the Danish cats was CMhm. Feline CMhm infections are often subclinical with only minor haematological changes [17, 22, 23] and in accordance with this, we found no significant difference in haematological values in cats being CMhm positive or negative.

This higher prevalence of CMhm carriage compared to the other haemoplasma species could be due to the lack of symptoms in these cats thus possibly allowing propagation and spread of the agent. Transmission between cats has been suggested, but not definitely demonstrated, to occur through biting wounds and from mother to offspring. Also haemoplasma DNA has been detected in the cat flea *Ctenocephalides felis* and ticks [12, 24]. Furthermore, these asymptomatic cats may impose a risk with the increased use of feline blood for transfusion. The only case of Mhf infection showed signs of haemoplasmosis with major haematological changes with a marked decreased erythrocyte count, and abnormal haemoglobin and haematocrit values. Furthermore the cat had an increased reticulocyte count and presence of nucleated erythrocytes. These findings supports the current view that Mhf is the most pathogenic species, and the one most often found associated with clinical disease [25].

We did not detect any CMt in the cats sampled in this study. Other prevalence studies have previously shown that CMt seems to be the least prevalent haemoplasma in Europe, ranging from 0 to 2.3% [12, 21, 26, 27]. In these studies, the sample sizes were between 3 to 20 times larger than our number of cats. A larger sample size could therefore be needed in order to conclude whether CMt is present in Denmark.

The significance of a positive PCR result should always be compared with clinical signs, pathological findings, haematological features and possible concurrent or complicating diseases. More severe clinical signs have occurred in cats experimentally dual-infected with Mhf and CMhm than in cats with mono-infection with either of the species and in cats spontaneously infected with both Mhf and CMhm [6, 28]. Based on PCR and sequencing of the PCR products, we found no samples with more than one haemoplasma species. The only CMhm positive cat in our study was diagnosed as FIV positive. The risk of infections with haemoplasma has previously been associated with concurrent diseases such as feline leukemia virus (FeLV) and FIV. Cats co-infected with FeLV develop a more severe anaemia than cats only infected with CMhm and FIV infection was shown to be associated with an increased risk of co-infection with CMhm and Mhf [7, 14, 29].

The risk of infection with feline haemoplasmas has been associated with different factors, such as age, gender, breed, environment, flea infestation and concurrent diseases [10–12, 15, 21]. Our results showed a statistical association between age and carriage of haemoplasma species, with an increased prevalence in older cats. These finding are consistent with findings in previous studies [12, 21, 26]. An association between risk of infection and increasing age may simply reflect a cumulative risk of exposure, since complete clearance of the organism once a cat has become infected has not been demonstrated. We also found higher prevalence of haemoplasma in domestic cats compared to purebred cats. Purebred cats are often held indoor, whereas domestic cats more often allowed being outdoor, where risk of exposure is higher.

## Conclusions

This is the first study to investigate carriage of feline haemoplasma among Danish cats. The study showed a prevalence of 16.4% with CMhm as the most prevalent species. Age and breed was significantly associated with haemoplasma carriage. These results are in accordance with similar studies from other parts of Europe, USA and Japan.

## Additional file

**Additional file 1: Table S1.** Sample characteristics of 67 cats, including age, gender, breed, symptoms and haemoplasma status.
