# Supplementary material for: Prevalence of feline haemoplasma in cats in Denmark
Source: Acta Vet Scand. 2016 Nov 10;58:78. doi: 10.1186/s13028-016-0260-1 (PMC5103603; doi:10.1186/s13028-016-0260-1)
Supplement: Supplementary file 1 — Additional file 1: Table S1. Sample characteristics of 67 cats, including age, gender, breed, symptoms and haemoplasma status. [file 13028_2016_260_MOESM1_ESM.docx]

**Additional file 1.** Sample characteristics of 67 cats, including age, gender, breed, symptoms and haemoplasma status.

| **Cat number** | **Breed** | **Gender** | **Age** | **Symptoms and/or diagnosis** | **PCR** | **RT-PCR** | **Species** |
| --- | --- | --- | --- | --- | --- | --- | --- |
| 1 | Persian | M | 4 y | Urolithiasis | - | - |  |
| 2 | Domestic | M | 9 y | Fever, depressed, slightly pale mucus membranes | + | + | CMhm |
| 3 | ? | M | ? | Persistent fever | - | - |  |
| 4 | Norwegian forest | F | 9 y | Anorexia, vomiting, weight loss | - | - |  |
| 5 | Domestic | M | 1 y 9 m | Kidney failure, uninar phlegmone | - | - |  |
| 6 | British shorthair | F | 6 y 11 m | Donated because of euthanasia | - | - |  |
| 7 | Domestic | M | 5 y 7 m | Anorexia, depressed, may be obstipated | - | - |  |
| 8 | Norwegian forest | M | ? | Fever, depressed, maybe anaemic | - | - |  |
| 9 | Domestic | M | 3 y | Hematuria, stranguria | - | - |  |
| 10 | Domestic | M | 9 y 8 m | Lymphocytotic lymphoma. Chemotherapy | - | - |  |
| 11=5 |  |  |  |  |  |  |  |
| 12 | Ragdoll | F | 6 y 11 m | Ventral bulla osteotomy | - | - |  |
| 13 | Domestic | M | 10 m | Dehydrated, bloody vomit, depressed | - | - |  |
| 14 | Domestic | M | 13 y | Alopecia | - | - |  |
| 15 | Maine Coon | M | 2 y | Obstipated | - | - |  |
| 16 | Domestic | F | 16 y | Anorexia, vomiting | - | - |  |
| 17 | Persian | M | 11 y | Chronic renal insufficiency | - | - |  |
| 18 | Norwegian forest | M | 3 y 8 m | Fever, dehydrated, depressed, anal bursitis | - | - |  |
| 19 | Domestic | F | 9 y | Depressed, obs.pro. FLUTD | - | - |  |
| 20 | Domestic | M | 17 y 11 m | Weight loss, control pad wound | - | - |  |
| 21 | Persian | F | 6 y 7 m | Oral plasmacytoma, control | - | - |  |
| 22 | Domestic | M | 10 y | Vaccination, teeth check, possible tooth cleaning | - | - |  |
| 23 | Domestic | F | 10 y 6 m | NAI | + | + | CMhm |
| 24 | Domestic | F | 7 y | NAI | - | - |  |
| 25 | Norwegian forest | M | 1 y 1 m | Blood drawing | - | - |  |
| 26 | Norwegian forest | M | 1 y | Blood donor screening | - | - |  |
| 27 | Domestic | F | 7 y | Urinary problem, gastrointestinal problem, lower respiratory problem | - | - |  |
| 28 | Burmese mix | F | ? | NAI | - | - |  |
| 29=10 |  |  |  |  |  |  |  |
| 30 | Norwegian forest | M | 11 y 11 m | Health check, possible polydipsia | - | - |  |
| 31 | Domestic | F | 16 y | Surgery for removal of mammary gland carcinoma | - | - |  |
| 32 | Domestic | F | 4 y | NAI | - | - |  |
| 33 | Domestic | M | 10 y 2 m | Limping, swollen paw. Amputation of toe | + | + | CMhm |
| 34 | Norwegian forest | M | 3 y 2 m | Blood donor screening | - | - |  |
| 35 | Domestic | F | 11y 3 m | Health check, vaccination | - | - |  |
| 36 | Birman | M | 3 y | NAI | - | - |  |
| 37 | Persian | M | 11 m | Hind part ataxia, obs. Pro. Juvenile hip joint dysplasia | - | - |  |
| 38=5 |  |  |  |  |  |  |  |
| 39 | Domestic | F | 12 y 9 m | Hyperthyroidism | - | - |  |
| 40 | Domestic | M | 11 y 3 m | Vaccination, deworming | + | + | CMhm |
| 41=10 |  |  |  |  |  |  |  |
| 42 | Domestic | M | 17 y | FIV positive, cardiac murmur, anorexia, depression, obs. Hyperthyroidism | + | + | CMhm |
| 43 | Maine Coon | ? | ? | NAI | - | - |  |
| 44 | Persian | F | 15 y | Kidney problem, cachexia | - | - |  |
| 45 | Domestic | F | 6 y | NAI | - | - |  |
| 46 | Domestic | F | 11 y | NAI | - | - |  |
| 47 | Norwegian forest | F | 13 y | NAI | - | - |  |
| 48 | Domestic | F | 8 y | NAI | - | - |  |
| 49 | European shorthair | F | ? | Spasms | - | + | CMhm |
| 50 | Domestic | F | 7 y | Staging of mammary neoplasia and following surgery removal | - | - |  |
| 51=4 |  |  |  |  |  |  |  |
| 52 | European shorthair | F | 8 y | Spasms, low blood glucose | + | + | CMhm |
| 53 | European shorthair | F | 2 y | Increased abdominal circumference, hair loss | - | - |  |
| 54 | Domestic | M | 4 y 10 m | Skin problems, obs. Allergy | - | - |  |
| 55=15 |  |  |  |  |  |  |  |
| 56 | European shorthair | F | 15 y | NAI | - | - |  |
| 57 | Domestic | F | 16 y | NAI | - | - |  |
| 58 | Domestic | F | 10 y 1 m | Bloody urine | - | - |  |
| 59 | Domestic | M | 4 y | NAI | - | - |  |
| 60 | Domestic | F | 12 y | NAI | - | - |  |
| 61 | Domestic | F | 8 y | Outdoor riam, weight loss, depressed, alopecia | - | - |  |
| 62 | Domestic | F | 16 y | Control hyperthyroidism | - | - |  |
| 63 | Domestic | F | 12 y | Mammae tumor | - | - |  |
| 64 | Domestic | F | 6 y | NAI | - | - |  |
| 65 | European shorthair | F | 14 y | Vomit containing yellow bile, eats and drinks normal. | - | - |  |
| 66 | Domestic | M | 14 y | Weight loss, anorexia, increased liver values | + | + | CMhm |
| 67 | Domestic | M | 2 y | Vomiting, weight loss | - | - |  |
| 68 | Domestic | M | 10 y | Referred patient larynx paresis sinister | + | + | CMhm |
| 69 | European shorthair | M | 10 y | Vomiting | - | - |  |
| 70 | Norwegian forest | M | 3 y 6 m | NAI | - | - |  |
| 71 | Maine Coon | M | 2 y 6 m | Chronically poor doer, weight loss | - | - |  |
| 72 | Domestic | M | 10 y | Azotemia, hypercalcemia (acute kidney failure), decreased appetite, depressed | + | + | CMhm |
| 73 | Domestic | M | 2 y 2 m | Weight loss, anorexia, dehydration | + | + | Mhf |
| Positive control for Mhf | | | | | + | + | Mhf |
| Positive control for CMhm | | | | | + | + | CMhm |
| Positive control for CMt | | | | | + | + | CMt |

NAI: No available information, Mhf: *Mycoplasma haemofelis*, CMhm: *Candidatus* Mycoplasma haemominutum, CMt: *Candidatus* Mycoplasma turicensis, FIV: feline immunodefiency virus, FLUDT: **Feline lower urinary tract disease,** Obs.pro.: Suspected to suffer from
